# Cellular phone and germ cell: A comment

**DOI:** 10.4103/0974-1208.63126

**Published:** 2010

**Authors:** Viroj Wiwanitkit

**Affiliations:** Department of Laboratory Medicine, Faculty of Medicine, Chulalongkorn University, Bangkok - 10330, Thailand

Sir,

The cellular phone is a widely used tool at present. The interesting question is whether cellular phone has any effects on germ cell. Basically, the effect of cellular phone on health is believed to due to microwave emitted from the phone. The effect of microwave from cellular phone on germ cell is a controversial issue.[1,2] The topic on health effect of cellular phone is of present interest.[3] Röösli *et al*. recommended that “a precautionary approach when dealing with radio and microwave frequency radiation is recommended for the individual and the general population.”[4] In this short paper, the author hereby briefly summarizes and comments on the recent reports on the effect of cellular phone on germ cell.

Focusing on the effect of cellular phone on ovary, according to the literature search, there is only one experimental report on this topic published by Gul *et al*.[1] They reached the conclusion that “microwaves of mobile phones might decrease the number of follicles in rats by several known and, no doubt, countless unknown mechanisms.”[1] There are some important considerations on the results of this work. First, this work had no matched control group and uses few numbers of subjects that might lead to some statistical unreliability. Second, the problem of preanalytical interference can be expected. It should be noted that the author did not monitor the background environmental microwave from external sources in the setting and also did not monitor this during the experiment. Third, whether they detected decreased number of the follicle is a physiological change of the rat during its estrous cycle is not well proved.

Focusing on the effect of cellular phone on testes, there are many reports on the effect of cellular phone on testicular cell. Effect of cellular phone on mammalian cells and sperm DNA as well as its effect on apoptosis is still controversial.[5] In an experimental study, microwaves emitted from a cell phone did not affect the testes of the studied rats.[2] However, similar criticisms on this kind of experiment as earlier discussed should be noted. For human model, Hardell *et al*. reported on null correlation between exposure to cellular phone and testicular cancer in their case control study.[6] However, there are many recent reports on *in vitro* studies pointing to the possible effect of cellular phone on human spermatozoa.[7–10] De Iuliis *et al*. reported that radiation from cellular phone might induce reactive oxygen species production and DNA damage in human spermatozoa *in vitro*.[7] Agarwal *et al*. also observed similar findings in their *in vitro* study and proposed that “keeping the cell phone in a trouser pocket in talk mode may negatively affect spermatozoa and impair male fertility.”[8] A decrease in the percentage of sperm cells in vital progressing motility in the semen relating to frequency of using mobile phones was also reported by Wdowiak *et al*.[9] However, these *in vitro* studies usually depend on small number of tested samples. This makes the limited implication of the results.

In conclusion, effect of cellular phone on the germ cell is still a myth. The previous reports usually pose some problems implying the need for further well-controlled studies for clarification on this topic.
